# GENDER DIFFERENCES IN SOCIAL JUDGEMENTS RELATE TO SOCIAL EXPERIENCE ON THE JOB FOR OLDER ADULTS

**DOI:** 10.1093/geroni/igz038.1119

**Published:** 2019-11-08

**Authors:** Cassandra Richards, Jennifer T Stanley

**Affiliations:** The University of Akron, Akron, Ohio, United States

## Abstract

Older adults (OA) are worse than young adults (YA) at recognizing emotional facial expressions (Ruffman, Henry, Livingstone, & Phillips, 2008). In particular, age differences in anger recognition remain even when other emotions improve via additional context (e.g., Richter, Dietzel, & Kunzmann, 2011; Stanley & Isaacowitz, 2015). We investigated whether job experiences with greater social components would relate to better anger recognition and interpersonal perception in OA. We expected OA who held jobs with more social requirements would be better at anger recognition and interpersonal perception, but that this would differ by gender. OA (N=194) reported their present job and completed an emotion perception task and the Interpersonal Perception Task-15 (IPT-15; Costanzo & Archer, 1989). Ratings from the O*Net database were used to determine the degree of social requirements (0-100) for reported jobs. For older females, more social experience in their present job was related to better anger recognition (r =.45,p=.014). More face-to-face experience in the job held the longest was related to better overall emotion perception in older females (r=.20,p=.047). For older males, more social experience in their present job was related to worse anger recognition (r=-.45, p=.029). More coordination and leadership experience in the job held the longest was related to better interpersonal perception in older males (r =.28, p=.010). These results suggest gender is important when examining the degree to which social experience in the workplace relates to social judgments. Future work should investigate whether gender differences in subordinate vs leadership roles can account for these findings.

